# The Amygdala and Politics

**DOI:** 10.3390/brainsci16070709

**Published:** 2026-06-30

**Authors:** Javier Díaz-Nido, Jesús Avila

**Affiliations:** 1 Centro de Biología Molecular Severo Ochoa, CSIC-UAM, 28049 Madrid, Spain; javier.diaznido@uam.es; 2 Departamento de Biología Molecular, Universidad Autónoma de Madrid, 28049 Madrid, Spain; 3 Centro de Investigación Biomédica en Red de Enfermedades Neurodegenerativas (CIBERNED), Instituto de Salud Carlos III, 28029 Madrid, Spain

**Keywords:** social relations, brain structure, amygdala, plasticity, cellular interactions, molecular interactions

## Abstract

Emotions play a central role in social interactions and associations, and they are regulated by multiple regions of the human brain. In this review, we focus primarily, almost exclusively, on the amygdala, highlighting functional and structural changes related to behavioral interactions that may occur within diverse social groups, including families, cultural associations, and political organizations, each typically structured around leaders and followers. More specifically, we examine political parties in democratic societies, after first outlining how the relationship between brain structure, particularly the amygdala, and behavior has evolved from non-human primates to humans, and how structural and behavioral changes may arise through aging or disease.

## 1. Introduction

After birth and during early development, animals must establish social relationships to ensure survival. The role of the mother and family is essential in this process. Humans, in particular, depend on prolonged social interaction for survival. This support is typically provided through positive interactions within a primary social group (the family) where the young individual acquires information and resources from other members, often within a system structured by rules and hierarchy [1].

In adulthood, beyond maternal care, non-human primates can form multilevel social systems, ranging from small, family-like groups, as observed in gibbons, to larger and more complex groups, such as those of chimpanzees [2]. Within these larger groups, individuals live under hierarchical structures, commonly patriarchal in chimpanzees [3], although matriarchal systems exist in some species, such as bonobos [4]. In such contexts, social interactions between leaders and subordinates are often shaped by dynamics of aggression and submission [5], and behaviors are strongly influenced by emotional responses related to survival.

From an evolutionary perspective, early human groups shared similarities with non-human primates, often exhibiting patriarchal structures that later evolved into more centralized forms of authority, such as autocracies led by dominant individuals [6]. As human societies expanded, driven by advances in knowledge transmission and technological innovation, larger social organizations emerged, including tribes and nations. In some cases, these systems transitioned from autocratic to democratic forms of governance, leading to the development of political parties [7]. In this way, changes in brain regions could correlate with behavioral differences. Although several brain regions may be involved, we focus on the amygdala, which is the most extensively studied brain structure in relation to political opinions.

Returning to primate social dominance behavior, the primary objective of subordinates is survival. In the transition from autocracy to democracy, additional goals emerge, including cognitive and cultural development aimed at achieving a higher quality of life. These behavioral differences may be associated with structural variations in the brain, a highly plastic organ, as Santiago Ramón y Cajal emphasized when introducing the concept of neuronal plasticity and noting that each individual may become the “sculptor of their own brain” [8,9]. Indeed, neuronal plasticity can give rise to multiple changes over time. Recently, topological turning points across the human lifespan have been reported [10]. Thus, changes in political attitudes and behavior may correlate with structural brain changes. To identify such changes, longitudinal brain imaging studies will be required, particularly to determine whether amygdala alterations precede political preferences or instead arise as a consequence of experiences, social environments, and interpersonal associations.

In the transition from primate social dominance to democratic behavior, the self-domestication hypothesis has been proposed [11,12,13], suggesting that an increase in positive social interactions over time may have facilitated improvements in quality of life. Adverse environmental conditions during early life can drive adaptive responses to stress that facilitate survival [14]. Such adaptations may result in behavioral traits favoring immediate rewards over long-term benefits [15]. Early-life environments have also been shown to shape the development of coping strategies and stress-related behaviors [16]. From an evolutionary perspective, brain structural changes that support positive social interactions and reduce stress exposure may have contributed to enhanced well-being and quality of life.

In democratic systems, rules are typically established to balance both collective and individual benefits, reflecting the preferences of the majority while allowing for periodic revision. Interestingly, the degree of democracy has been associated with certain societal characteristics, including higher levels of benevolence, lower levels of malevolence, and greater overall well-being [17]. Conversely, a negative association has been observed between malevolent creativity and experiences of childhood neglect [18].

## 2. Results

### 2.1. Social Behaviors Types

The pioneering work of Panksepp (see reference [19]) demonstrated that feelings are a key component of affective science and are closely linked to social interactions. Panksepp described both positive and negative emotions [20,21]. These emotions are associated with specific brain regions; for example, the amygdala is widely recognized as a central neural hub for emotional processing through its extensive connections with other brain areas. Focusing on the amygdala, Panksepp noted [20] that negative emotions such as fear are accompanied by increased amygdala activity.

In this review, we will later address several themes related to social interactions and behavioral types, including brain structures involved in emotional and behavioral plasticity, with particular emphasis on the amygdala. We will also discuss the potential relationship between brain structure and function, the role of the amygdala in shaping political ideology, and factors such as disease or aging that influence amygdala structure and function at the cellular and molecular levels.

In democracy, behaviors within social groups are generally classified into two main types: conservative (traditional) and liberal (progressive). This dichotomy is particularly evident in the British and American contexts, where two major parties typically represent these opposing political orientations. However, the picture becomes considerably more complex within the multiparty and multidimensional political landscapes characteristic of most European countries. Previous studies have reported significant psychological differences between individuals with divergent political ideologies [22] and have even suggested a potential genetic basis for these distinctions [23]. In light of these findings, it can be hypothesized that genetic factors may influence neurodevelopmental processes, leading to distinct brain configurations that predispose individuals toward specific political preferences. Consequently, an important question emerges: does political ideology correlate with brain structure changes?

Different brain areas may influence human behavior; however, in this review we focus on the amygdala [24,25,26], which may play a role with the types of social interactions observed within particular political parties [27]. However, findings across studies may vary depending on methodological differences, including how amygdala volume is measured, for example, whether analyses focus on the entire structure or on specific subregions (e.g., left versus right amygdala) [28,29], as well as whether gender differences among participants are taken into account [30].

### 2.2. Social Relations: Association of Political Ideology with Brain Structure: The Role of Amygdala

In a pioneering study conducted in Great Britain, political orientations were correlated with brain structure in young adults [31]. Left-wing attitudes were associated with greater gray matter volume in the anterior cingulate cortex (ACC), whereas right-wing attitudes were linked to increased gray matter in the amygdala [31]. These findings suggest that the distinct ideologies of conservative and liberal parties, particularly in their economic and social dimensions, may relate to differences in brain regions involved in decision-making, such as the ACC, and emotional processing, such as the amygdala [31]. Indeed, the amygdala plays a crucial role in processing emotions such as elation, reward, sadness, and fear, which in turn help regulate our social behaviors [32,33,34,35].

A subsequent study conducted in Singaporean Chinese adults also found a significant positive correlation between right-wing attitudes and the bilateral amygdala, alongside a negative correlation with the ventromedial prefrontal cortex [36].

Efforts to replicate these findings in the more complex multiparty Dutch political context confirmed the correlation between increased amygdala volume and conservatism and identified a novel link between the volume of the right fusiform gyrus and conservative ideology. However, the previously reported association between greater gray matter in the ACC and left-wing ideology was not replicated [37]. These results highlight the particularly intriguing role of the amygdala in shaping political preferences.

Comparative studies examining political orientations among patients with frontal lobe or amygdala lesions, as well as healthy controls, revealed that greater damage to the dorsolateral prefrontal cortex (DLPFC), but not to the amygdala, was associated with stronger conservative attitudes [38]. Interestingly, repetitive transcranial magnetic stimulation of the DLPFC has also been linked to an increase in conservative values [39]. Due to its deep anatomical location, however, there are currently no studies using non-invasive stimulation of the amygdala. More recently, a negative correlation between right-wing authoritarianism and the volume of the dorsomedial prefrontal cortex has also been reported [40]. Collectively, these findings suggest that both the amygdala and prefrontal regions contribute to the modulation of political attitudes.

A larger amygdala volume has been associated with emotional states such as sadness and fear [37,41]. Additionally, individuals with larger amygdalae are less likely to participate in political protests [42], possibly reflecting the amygdala’s role in perceiving and maintaining social hierarchies [43]. Consequently, a larger amygdala may predispose individuals to view conservative social structures as legitimate and desirable [44]. Conservative ideologies are often linked to family or cultural traditions that emphasize stability and hierarchy [45], whereas right-wing political views are typically associated with traditionalism and resistance to social change. In contrast, left-wing ideologies are linked to egalitarianism and efforts to transform society to promote equality [46]. Furthermore, given that political views can evolve throughout an individual’s life, such longitudinal studies would help determine whether shifts in ideology are accompanied by structural changes in the amygdala or other regions, such as the prefrontal cortex (PFC) and ACC, implicated in executive function and decision-making [47].

### 2.3. The Role of the Amygdala in the Configuration of Political Ideology

Human survival is largely supported by positive social interactions, which occur within family, friendship, cultural, or political groups. These ‘in-groups’ are structured by distinct rules that can render interpersonal relationships either harmonious or conflictual. Interactions between high- and low-ranking members, such as those observed in political parties, are often the most complex and potentially conflictive.

As previously described, amygdala volume may correlate with the recognition of social hierarchy observed in followers of conservative parties. It has been proposed that low-rank followers of these parties, often described as honest individuals, strongly attached to family-like traditions, tend to show a larger amygdala volume [37]. As noted above, although other brain regions beyond the amygdala may also contribute to the recognition of social and political behavior, in this article we focus specifically on the role of the amygdala.

**Figure 1:**
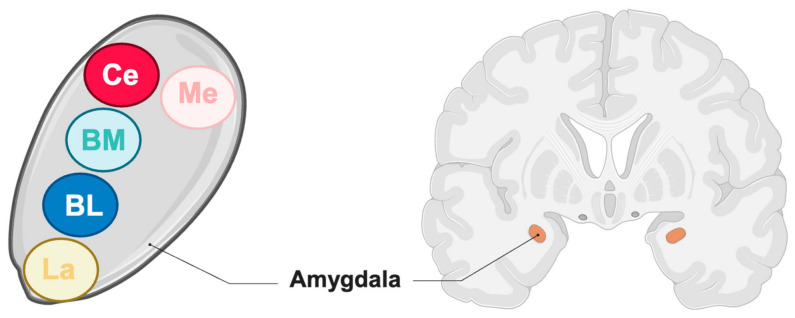
Localization of the amygdala within the brain (**right**) and schematic representation of selected amygdala nuclei (**left**), including the central nucleus (Ce), medial nucleus (Me), basomedial area (BM), basolateral area (BL), and lateral area (La). The central nucleus (Ce; red) projects to the hypothalamus, whereas the basolateral area (BL; blue) connects with the prefrontal cortex (PFC).

The amygdala is a complex structure composed of at least 13 nuclei, including the central nucleus, medial nucleus, and the basolateral complex, which encompasses the basomedial, basolateral and lateral area nuclei [48] (Figure 1). This structure plays a critical role in emotional processing and motivated behavior [49], including functions related to fear, aggression, and anxiety [50]. Importantly, the human amygdala undergoes both structural and functional changes across development [50,51].

In this review, we consider studies that employed different methodologies to address similar questions, such as variations in amygdala size. Although some differences were observed, the results consistently indicated similar trends [28,29,41], likely due to the use of comparable protocols. For instance, anatomical studies assessing amygdala volume often utilize structural Magnetic Resonance Imaging (MRI) [52,53]. Conversely, functional investigations can employ functional magnetic resonance imaging (fMRI) (34) or event-related potentials (ERP) measured via electroencephalography (EEG) electrodes to detect electrical changes in response to sensory or cognitive events [54].

The reported data indicate that responses to external social stimuli are faster and more immediate in the right amygdala, whereas the left amygdala is primarily involved in the cognitive aspects of emotion, enabling detailed evaluation and processing of both positive and negative emotional information [29,41]. Accordingly, the right amygdala plays a key role in the rapid detection of negative emotions, such as fear, and has been reported to be larger in individuals with conservative political orientations [55,56]. In this context, functional connectivity between the right amygdala and the left insula has been proposed [57,58].

Moreover, gender-dependent differences in amygdala function have been described, with the left amygdala more frequently activated in women [28]. In women, amygdala activation appears to be more closely associated with internal emotional processing, whereas in men it is more strongly linked to externally directed behavioral responses [30,53,59].

The amygdala engages in multidimensional processing through extensive connectivity with brain regions involved in sensory integration, reward, visceral responses, memory, emotional valence, and decision-making [60]. Its functional organization reflects the specialization of distinct nuclei [61]. In the context of political preferences, nuclei involved in visceral reactions—such as the central nucleus [62], and those implicated in decision-making, such as the basolateral amygdala [61], may be of particular relevance. These systems may mediate responses ranging from instinctive “fight-or-flight” reactions to more nuanced, flexible, or pragmatic behaviors [60], potentially corresponding to more liberal or conservative behavioral tendencies.

The amygdala also functions as a central node in the perception of social hierarchy, as shown in animal models. For example, studies in mice have demonstrated that serotonergic neurons of the dorsal raphe nucleus projecting to the central amygdala modulate social dominance behaviors [43,63].

Experimental models further support the remarkable neuroplasticity of the amygdala. For instance, exposure of a smaller mouse to a larger, dominant conspecific can trigger fear responses that promote the formation of new synaptic connections between specific engrams and amygdala neurons [64].

### 2.4. Emotions and Amygdala

Amygdala activation may therefore arise from fear induced by dominant social hierarchies. This activation engages amygdala–hypothalamic pathways, triggering stress responses that mediate the “fight-or-flight” reaction to adversity [60]. Two main physiological pathways are involved. One is controlled by the sympathetic–adrenal–medullary (SAM) system and leads to the release of noradrenaline. The other involves activation of the hypothalamic–pituitary–adrenal (HPA) axis, resulting in chronic stress and sustained cortisol release [65]. Also, the amygdala is capable of coordinating adaptive behavioral responses to threats [66]. Through amygdala–prefrontal connectivity [67,68], individuals may sustain social relations even under adverse or hierarchical conditions.

**Figure 2:**
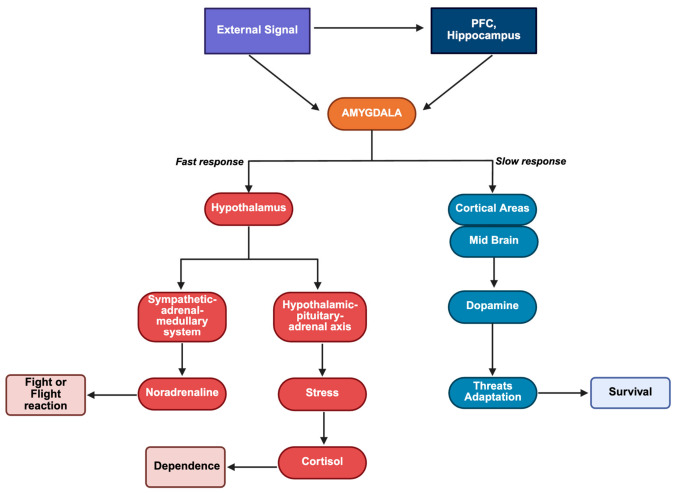
Differential amygdala connectivity with the hypothalamus and the prefrontal cortex. Amygdala–hypothalamic connections are associated with stress responses and hormone secretion, including noradrenaline and cortisol, whereas amygdala–prefrontal cortex (PFC) connections support threat-adaptation-based behaviors and the modulation of neurotransmitter systems such as dopamine. These connectivity patterns may be associated with progressive or conservative attitudes.

One possible adaptive route involves amygdala–prefrontal connectivity [67,68] (Figure 2), which may support the maintenance of social relationships even under adverse or hierarchical conditions. In this framework, the PFC contributes to adaptive behavior, as demonstrated in mice through the activation of dopaminergic projections [69]. Dopamine neurons in the ventral tegmental area (VTA) encode reward-prediction errors that guide learning by influencing projections to the striatum and PFC, thereby enabling new reward associations and behavioral adjustments [69].

Decision-making processes may also involve a balance, or “tipping”, between fairness, associated with temporal cortex activity, and efficiency, more closely linked to the PFC [70]. In this way, the recognition and acceptance of a social hierarchy could, paradoxically, facilitate social cohesion and interpersonal interactions.

Furthermore, mirror neurons [71], located in the premotor cortex [72], may also contribute to intrasocial interactions by enhancing empathy and emotional resonance among political interlocutors, further supporting cooperative behavior within hierarchical structures.

In summary, as illustrated in Figure 2, life stress exerts persistent and widespread effects on the prefrontal cortex–hypothalamus–amygdala circuit, as well as on dopaminergic pathways. These effects are mediated, at least in part, by alterations in the hypothalamic–pituitary–adrenal (HPA) axis and may contribute to the development of psychopathological disorders [73]. Indeed, depressive states have been associated with structural changes in the brain. More recently, the combined analysis of brain structural changes and proteomic biomarkers has provided a more comprehensive view of the biological mechanisms underlying depression and related psychiatric disorders [74].

### 2.5. Amygdala Plasticity in Behavioral Changes Due to Social Interactions Across the Lifespan

Age-related behavioral changes are associated with both structural and functional modifications of the amygdala. During development, the amygdala increases in size from birth through adulthood, in contrast to the hippocampus, which also increases [75] but follows a different developmental trajectory [76,77].

In later life, amygdala volume tends to decrease with aging; however, this reduction appears to be attenuated in individuals who maintain good social interactions [27].

Regarding behavioral influences, traumatic experiences resulting from negative social interactions can induce heightened activation of the central and basolateral nuclei of the amygdala [78]. Moreover, synaptic plasticity within amygdala networks underlies the acquisition, expression, and extinction of conditioned fear responses [79]. In contrast, positive social interactions do not seem to accelerate age-related reductions in amygdala volume and may even exert a protective effect against such decline [27].

The combined Impact of age and social interaction on amygdala structure and function has been examined in several contexts, including peripubertal stress [80], socio-emotional competencies in preterm youth [81], and exposure to maternal depressive symptomatology from birth in 10-year-old children [82]. In these conditions, a larger amygdala volume has frequently been reported. Notably, as mentioned above, positive social interactions appear to mitigate age-related amygdala volume reduction during aging [27].

In addition to structural alterations, different types of social interactions are also associated with functional changes in the amygdala, primarily mediated by synaptic plasticity mechanisms [53,83,84].

### 2.6. Behavior and Amygdala Plasticity: Autism, and Political Preferences

With respect to social interaction, autism spectrum disorder (ASD) is a neurodevelopmental condition characterized by marked alterations in social behavior and communication [85]. Neuroimaging studies using functional magnetic resonance imaging have revealed alterations in amygdala activity in individuals with ASD, giving rise to the “amygdala theory of autism,” which proposes that reduced amygdala activity contributes to the core social deficits observed in this condition [86].

As noted earlier, amygdala volume typically increases from birth through adulthood; however, this developmental enlargement is not observed in adolescents with ASD [76,77]. In adulthood, individuals with ASD continue to show atypical amygdala structure and function compared with neurotypical populations.

Interestingly, survey data from the United States indicate that a majority of adults with ASD who are registered to vote tend to affiliate with the Democratic Party and identify as politically liberal [87]. While the mechanisms underlying this association remain unclear, it suggests a possible link between social cognition, neural substrates, and political preferences may exist, although it is also important to acknowledge the potential influence of psychosocial factors in this relationship.

As discussed later in this article, molecular alterations involving proteins such as tau (see, for example, reference [88] for an overview) have been implicated in several neurodevelopmental and neurodegenerative disorders, including ASD [89].

### 2.7. The Relationship of the Amygdala with Social Interactions Based on Hierarchies

Ethological studies indicate that the establishment of social hierarchies is essential for maintaining stability within social groups across multiple species, including fish, rodents, and primates. Interestingly, behavioral and physiological changes associated with rising or falling within a hierarchy can occur over short periods, suggesting that these social states are remarkably plastic [90]. Several brain regions, including the amygdala, contribute to these changes.

The formation of dominance hierarchies involves social conflicts, accompanied by increased activity in the habenula–interpeduncular system among higher-ranking individuals. This enhanced activity correlates with aggressive behavior reminiscent of the “alpha male” model [91]. Similarly, in mice, variations in amygdala volume have been observed across individuals occupying different social ranks [43].

In murine models, other brain regions such as the habenula also exhibit structural changes in dominant individuals. Interestingly, these changes may reverse following the loss of hierarchical status, which can result in a depressive-like behavioral state [92].

Oxytocin (OT), a neuropeptide closely linked to amygdala function, also plays a crucial role in regulating social behavior. OT enhances amygdala–prefrontal cortex connectivity, thereby modulating emotional regulation [93]. In animal studies, OT has been shown to reduce amygdala activity and promote social interactions [94]. In humans, oxytocin’s positive influence on prosocial behavior, particularly within familial and close group contexts, is well documented. However, its potential role in promoting ethnocentric or in-group-biased behavior remains less understood [95]. Beyond its common characterization as the “love hormone,” oxytocin can also facilitate defensive or even aggressive responses toward perceived outsiders, especially in individuals with low anxiety levels [96].

### 2.8. Are There Differences in Brain Structure Between Leaders and Followers of Political Parties?

The critical role of the amygdala in establishing and modulating social hierarchies raises the question of whether differences exist in amygdala volume or activity between political leaders and their followers. In humans, amygdala volume has been shown to correlate with the size and complexity of an individual’s social network, although it does not appear to be related to other social variables such as life satisfaction or perceived social support [27]. To date, no experimental studies have directly addressed this question in humans, so insights must be drawn from animal models, with careful speculation about their relevance to human social structures. Thus, more dedicated empirical studies will be required before stronger conclusions can be drawn.

Using magnetic resonance imaging, a larger and more highly connected amygdala, particularly in relation to the raphe nucleus and hypothalamus, has been reported in dominant animals [97]. In mice, high-ranking individuals exhibit structural changes in areas such as the habenula, which can reverse when these mice lose their dominant status, often resulting in depressive-like behaviors [92]. In rats, high-ranking individuals display stress resilience without amygdala changes, whereas low-ranking rats show submissive behaviors accompanied by increased dendritic spine density in the amygdala [98].

These findings prompt the question of whether social rank within human political parties, and access to power, might similarly influence the architecture of brain regions, including the amygdala. In humans, factors beyond ideological orientation, such as the duration and level of power held by a leader, are crucial. Power can be defined as the ability to influence the actions, beliefs, or behavior of others within a social group [99,100]. Leaders wielding absolute or long-term power may transform social ideologies into personal agendas, increasing the risk of corruption. Historical and contemporary evidence supports this link: absolute power can lead to corruption [101], and extended tenure in power can amplify this risk [102]. As Lord Acton famously noted in 1887: “Power tends to corrupt, and absolute power corrupts absolutely.” So, the connection between corruption and politics has a long and well-documented history. Interestingly, research has shown a correlation between lower amygdala activation and a greater propensity to adapt to dishonest behavior [103], which may help explain the so-called “slippery slope” of corruption. Additionally, evolutionary game theory models suggest that social evolution can contribute to the persistence of corrupt behaviors [104]. In the political arena, corruption may be further facilitated by the creation of new norms or rules designed to extend the tenure of corrupt governments, as predicted by mathematical models of norm evolution in social judgment [105].

Reduced amygdala activation has been correlated with greater adaptation to dishonest behavior [103], potentially explaining the so-called “slippery slope” of corruption [103]. Evolutionary game theory models further indicate that social evolution may naturally give rise to persistent corruption [104]. In political contexts, corruption may also be reinforced by the creation of new norms that sustain governance structures favorable to long-term incumbency [105].

### 2.9. Factors Affecting Political Preferences and Amygdala Changes: Aging and Disease

#### 2.9.1. Aging

Aging is associated with alterations in amygdala functional neuronal connectivity, as revealed by neuroimaging studies [106]. Specifically, older adults show decreased connectivity between the amygdala and hippocampus but increased connectivity between the amygdala and dorsolateral prefrontal cortex [106]. These changes correlate with age-related reductions in memory for negative stimuli and a general enhancement of emotional memory, producing a “positivity effect” in older adults, a diminished processing of negative stimuli relative to positive ones compared with younger adults.

The positivity effect has been interpreted through the cognitive control hypothesis, which suggests that older adults engage prefrontal cortex activity more actively to regulate emotions during tasks involving emotion processing [107].

#### 2.9.2. Pathological Aging

Aging is the principal risk factor for several neurodegenerative disorders, including AD. Moreover, the development of AD also accelerates pathological aging processes [88].

The amygdala is also involved in the pathogenesis and progression of neurodegenerative disorders, including tauopathies such as Alzheimer’s disease (AD). Tau is a microtubule-binding protein [108] that, under pathological conditions, can undergo abnormal aggregation [88,109]. In the tauopathy known as frontotemporal dementia with parkinsonism linked to chromosome 17 (FTDP-17), disinhibition associated with amygdala dysfunction has been reported [110]. In AD, preclinical tau deposition in the amygdala has been linked to altered functional connectivity and early mood changes [111]. Early tau deposition correlates with reduced amygdala volume in amyloid beta-positive (Aβ+) clinically normal individuals, suggesting preclinical AD changes [112,113]. More detailed analyses indicate that atrophy in specific amygdala subnuclei, cortical, central, medial, and accessory basal, is associated with tau accumulation in Aβ+ individuals, even when total amygdala volume appears unchanged [112,113]. In familial autosomal dominant AD (FAD), tau accumulation begins in the basal nucleus, with basal and lateral nuclei linked to early cognitive deficits and depressive symptoms [114].

Changes in the amygdala have also been linked to a wide range of pathological conditions [115]. It may serve as a key site where relatively benign clinical states transition to more aggressive disease in disorders with multiple misfolded proteins, such as tau, α-synuclein, and TDP-43 [116]. Beyond AD [117], amygdala atrophy has been linked to epilepsy [118] and Parkinson’s disease (PD) [119]. Notably, two subtypes of PD have been proposed: body-first, originating in the periphery, and brain-first, which initiates in the central nervous system (CNS) and prominently affects the amygdala [120]. It would be intriguing to investigate whether political attitudes differ in patients with the brain-first subtype, potentially in association with amygdala changes.

Amygdala dysfunction has been implicated in temporal lobe epilepsy [121]. Consequently, electrical stimulation of the amygdala has been employed as a therapeutic approach in some patients with this condition [122]. As a result of such stimulation, the elicitation of distinct emotional responses has been observed. Specifically, stimulation of the left amygdala has been associated with both positive and certain negative emotions, whereas stimulation of the right amygdala predominantly evokes negative emotional states [123].

#### 2.9.3. Amygdala and Disease

Human lesion studies have been invaluable in delineating brain functions. Data indicate that a network of regions, including the amygdala, contributes to the regulation of emotional intelligence [124]. Recent evidence highlights the role of the basolateral amygdala in modulating altruistic versus selfish behavior: patients with Urbach–Wiethe disease and isolated bilateral damage to this region were less generous and exhibited steeper social discounting than healthy individuals [125].

Nevertheless, pioneering work on disorders closely associated with amygdala degeneration emerged from the clinical study of patient S.M., who was affected by Urbach–Wiethe disease. This rare disorder, previously mentioned, leads to bilateral amygdala damage and is characterized by a profound absence of fear, while other emotions such as happiness and anger remain largely preserved [126,127].

In addition, reduced amygdala volume has been reported in individuals exhibiting psychopathic traits, which are associated with diminished fear and empathy and increased aggression [128,129]. However, the relationship between amygdala structural and functional features and psychopathy remains complex and warrants further investigation [130].

#### 2.9.4. Amygdala Changes at Cellular Level

Like other brain regions, the amygdala is composed of both neurons and glial cells. Consequently, alterations in the amygdala may reflect changes in neurons, glia, or both, and these changes can be either structural or functional. Functional modifications, particularly those involving synaptic interactions, occur primarily at the neuronal level. Multiple neuronal subtypes have been characterized in the mouse amygdala [131].

A relatively high glia-to-neuron ratio has been reported in the amygdala [132]; however, this ratio appears to be reduced in individuals with major depressive disorder (MDD) [133]. This observation suggests that glial cells may play a central role in structural changes of the amygdala, such as atrophy or enlargement. Supporting this idea, the glia-to-neuron ratio in the amygdala is decreased in MDD-associated atrophy [133,134]. Interestingly, this reduction can be mitigated by treatments with antidepressants such as lithium or valproate, which have been proposed as protective agents against amygdala atrophy.

A low glial cell density is also observed in the amygdala of patients with MDD, with reductions primarily affecting oligodendrocytes [135], although other glial subtypes are also decreased [136]. The impact of stress on glial populations in the amygdala has been further investigated, highlighting the sensitivity of these cells to environmental and physiological challenges [137].

#### 2.9.5. Amygdala Changes at Molecular Level: Role of Tau in Amygdala

As previously indicated, the present work has primarily focused on the role of the amygdala in emotionally induced behavior; however, relatively little attention has been given to the molecular and cellular mechanisms underlying amygdala function and dysfunction in neurodegenerative disorders, beyond the previously discussed role of tau in autism [89]. Notably, the amygdala is critically involved in the pathogenesis and progression of neurodegenerative diseases such as Alzheimer’s disease (AD).

Looking for a possible mechanism underlying tau pathology in the amygdala, we can propose that negative emotions such as fear may activate the amygdala [138], leading to chronic stress (see Figure 2), a condition known to exacerbate tau pathology [139,140,141].

More recently, atrophy affecting several amygdala nuclei has been associated with mild cognitive impairment, a prodromal stage of Alzheimer’s disease [142].

In recent years, there has been a renewed interest in the analysis of the amygdala in AD [117], following the pioneering work of Braak and Braak [143]. Their studies demonstrated that tau pathology, one of the hallmark features of AD, is present in the amygdala during the earliest stages of disease progression.

More recently, tau positron emission tomography (tau-PET) studies have shown that tau accumulation in specific amygdala subregions, particularly the basal amygdala, is associated with cognitive impairment and neurological symptoms in carriers of familial Alzheimer’s disease mutations [114]. In addition, tau deposition in the amygdala of older apolipoprotein E4 (ApoE4) carriers has been linked to depressive symptoms [144]. Similarly, elevated tau levels in the medial amygdala have been associated with mood-related symptoms in individuals with preclinical AD [111].

**Figure 3:**
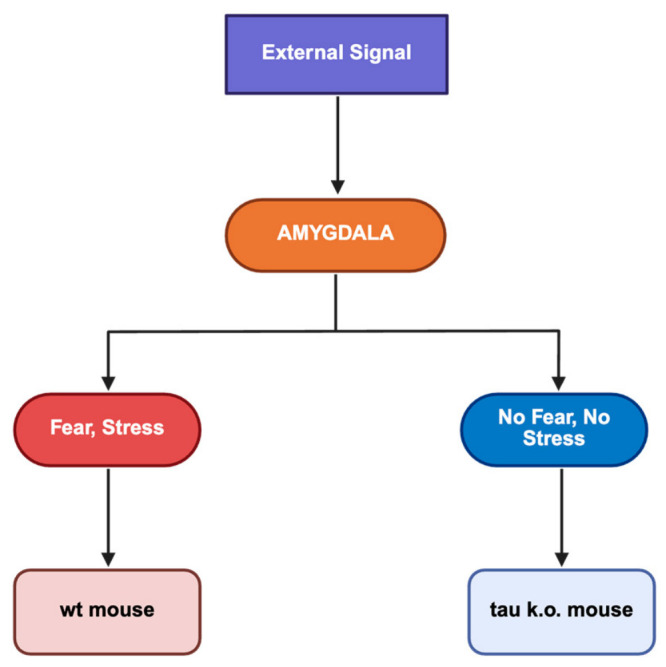
A relationship between chronic stress and tau accumulation has been proposed [140]. In addition, alterations in amygdala function have been reported in a mouse model of tauopathy characterized by high levels of tau accumulation [110]. Emotional behavioral abnormalities, including altered fear-related responses, have also been described in tau knockout (tau k.o.) mouse models [146].

Several of these observations in humans have been replicated in mouse models of tauopathies [110,140,145], where a close relationship between stress conditions and tau pathology has been described. Although tau accumulation may, in part, arise as a consequence of chronic stress, evidence from tau knockout mice indicates a markedly attenuated response to fear-induced stress, suggesting a direct role for tau in amygdala-mediated emotional processing [146] (Figure 3). For future research, we propose analyzing specific amygdala nuclei, cellular populations, and molecular mechanisms, together with social and political behaviors, that may contribute to individual differences.

## 3. Discussion

It has been proposed, with the indicate methodological limitations, that the amygdala plays a central role in shaping human behavior [122]. During evolutionary development, particularly in primates and likely from prehistoric humans to modern Homo sapiens, an expansion of the amygdala complex appears to have occurred [11]. Functionally, this expansion may have facilitated a transition from rapid, reflexive decision-making processes toward more regulated and slower evaluative responses.

The self-domestication hypothesis suggests that one of the most significant changes during human evolution was emotional in nature: humans became more tolerant of one another and markedly less aggressive or fearful in social contexts [11,12,13]. A reorganization of the amygdala may have played an important role in this process, given the centrality of emotions in social interactions, including those related to political behavior.

Both structural and functional plasticity occur within the amygdala. Amygdala volume increases from birth to adulthood, accompanied by an increase in neuronal and glial cell numbers; however, this developmental expansion is attenuated in disorders such as autism spectrum disorder [76]. In addition, socioeconomic status appears to play an important role in determining amygdala volume, with smaller volumes reported in individuals from lower socioeconomic backgrounds [147].

Adverse environmental conditions and negative social relationships may further impair amygdala growth, whereas improvements in quality of life may help prevent volume reduction, similar to what has been described during aging in older individuals with sustained positive social interactions. During adulthood, chronic stress is associated with amygdala atrophy, which may correlate with elevated cortisol levels and involve not only neuronal loss but predominantly glial cell loss. Finally, during aging, a time-dependent decrease in amygdala volume occurs; however, as noted above, positive social interactions may attenuate this age-related decline.

Evidence indicates that individuals with conservative political views tend to have larger amygdala compared with those holding left-wing attitudes [37]. However, whether differences exist in the amygdala between political leaders and their followers remains unclear. We hypothesize that leaders with high social status and prolonged power may be at increased risk of corruption, which could be associated with decreased amygdala volume, but more data are needed to reach those conclusions. Consequently, research should focus not only on followers but also on political leaders to explore this potential association.

Previous studies linking larger amygdala volume to conservative views [31,37] have important limitations. The amygdala is highly heterogeneous, comprising at least 13 distinct nuclei, making overall volume a relatively crude measure. Future studies should aim for higher-resolution analyses that can distinguish subnuclear differences. Additionally, the amygdala is dynamic across the lifespan, undergoing changes from adolescence to adulthood [148] and in response to chronic stress [149]. The presence of a larger amygdala in young adults with conservative views may indicate a causal role in shaping conservatism, but longitudinal studies are essential to determine whether amygdala size drives political orientation or vice versa [31].

Another question that should be addressed is the cellular base of these differences in amygdala size in people with different political orientations, with a particular attention to glial cell density. This might easily be approached through the histochemical analysis of postmortem tissue, but there are no records about political attitudes of donors in brain banks. Recent advancements in neuroimaging techniques like diffusion MRI might allow the non-invasive mapping of glial density in the living human brain [150,151].

Future research in political neuroscience should also examine the functional connectivity of the amygdala and its subnuclei with other brain regions implicated in political ideology. For instance, a recent study found that activity in the amygdala, inferior frontal gyrus, and hippocampus was strongly associated with political affiliation [152]. A deeper understanding of these networks could shed light on the neural mechanisms underlying political behavior and decision-making.

For a more rigorous analysis, future research should integrate neuroimaging not only with cellular and molecular approaches but also with social, cultural, developmental, and socioeconomic variables, although bringing together research groups with such diverse expertise is not always an easy task.

In summary, studies on the evolution of social interactions from non-human primates to modern humans suggest a transition, in many societies, from predominantly patriarchal hierarchical structures to democratic systems, although autocratic forms of governance persist in some regions. This transition toward democracy has been associated with a greater prevalence of benevolent traits among citizens [17], who may also display increased tolerance in their social interactions [11,12,13].

At the neurobiological level, efforts to relate these evolutionary changes to brain structure have reported an expansion in amygdala size from non-human primates to humans. This observation has been tentatively linked to behavioral tendencies associated with conservatism, such as risk aversion and the preservation of existing social status. In contrast, more liberal orientations, often associated with a relatively smaller amygdala, may involve greater openness to novelty, knowledge acquisition, and social change.

Thus, based on evolutionary changes in the brain that are thought to promote more tolerant behavior among members of social groups, often framed within the self-domestication hypothesis [11,12,13], one might anticipate increasingly positive social interactions over time. However, an important caveat remains. This concerns the brain structure and function of group leaders, particularly within political contexts. Addressing this gap will require more systematic investigation into the neurobiological characteristics of leaders compared with followers.

## Data Availability

We are open to share the comments (and hypothesis) indicated in this article with the readers of this review.

## Abbreviations

The following abbreviations are used in this manuscript:

ACC	Anterior Cingulate Cortex
DLPFC	Dorsolateral Prefrontal Cortex
PFC	Prefrontal Cortex
fMRI	Functional Magnetic Resonance Imaging
ERP	Related Potentials
EEG	Electroencephalography
SAM	Sympathetic Adrenal Medullary
HPA	Hypothalamic–Pituitary–Adrenal
VTA	Ventral Tegmental Area
ASD	Autism Spectrum Disorder
OT	Oxytocin
AD	Alzheimer’s Disease
Aβ+	Amyloid Beta Positive
FAD	Familial Autosomal Dominant Alzheimer Disease
PD	Parkinson’s Disease
CNS	Central Nervous System
MDD	Major Depressive Disorders
Tau-PET	Tau Positron Emission Tomography
